# Social network site use and materialistic values: the roles of self-control and self-acceptance

**DOI:** 10.1186/s40359-024-01546-7

**Published:** 2024-01-30

**Authors:** Qing Yang, Ying Xu, Kees van den Bos

**Affiliations:** 1 School of Psychology, Qufu Normal University, Qufu, China; 2 CAS Key Laboratory of Mental Health, Institute of Psychology, Chinese Academy of Sciences, Beijing, China; 3 Department of Psychology and School of Law, Utrecht University, Utrecht, Netherlands

**Keywords:** Social network site, Materialistic values, Materialism, Self-control, Self-acceptance

## Abstract

**Background:**

While prior studies have established a close association between the use of social network sites (SNSs) and materialistic values, there is limited understanding of the mediating and moderating mechanisms related to important self-related processes, such as self-control and self-acceptance. This paper explores whether and how these factors play a role in comprehending online behavior. One could state that frequent SNS use may pose a risk of virtual addiction, may be related to decreased self-control capacity, and may increase attention to material information on SNS, thereby making it more likely that users affiliate with behaviors associated with materialistic values. In contrast, self-acceptance, as a stable self-process indicating a genuine alignment with one’s true self and the ability to make decisions based on inner needs, may be related with reduced engagement in complex information on SNSs. Consequently, this could serve as a buffer against excessive SNS use and its potential associations with issues of self-control and materialistic values.

**Methods:**

A total of 706 Chinese college students were surveyed in a cross-sectional study. They completed self-report questionnaires including the WeChat use intensity scale, the Material Value Scale, the Trait Self-control Scale, and the Self-acceptance Questionnaire. A moderated mediation model was examined to test predictions.

**Results:**

SNS use intensity was positively associated with materialistic values, and self-control partially mediated this association. That is, higher intensity SNS users are more likely lower in self-control, which relates to stronger materialistic values. In addition, the indirect effect through self-control was moderated by self-acceptance, such that this indirect effect was significant only for individuals with low levels of self-acceptance.

**Conclusions:**

This study reveals that self-acceptance may be a protective factor that helps to mitigate excessive SNS use and its potential effects on self-control and materialistic values. It further suggests that psychological interventions targeting the enhancement of self-acceptance and self-control could hold promise in alleviating the negative association between SNS use and materialistic values.

**Supplementary Information:**

The online version contains supplementary material available at 10.1186/s40359-024-01546-7.

## Introduction

Materialistic values exert a nuanced influence on modern social values and lifestyles, largely due to the flourishing of consumer culture and capitalism. Commence with classical literature, exemplified by Fromm, who posited that contemporary society is primarily steered by a materialistic orientation fixated on amassing possessions and external accomplishments (having), often overlooking spiritual and emotional contentment (being) [1]. Veblen’s theory of the leisure class proposed that the quest for material possessions and conspicuous consumption becomes a defining trait of the leisure class, shaping their social status, and influencing society at large to embrace materialistic values [2]. With the swift pace of modernization and economic growth, materialism and consumerism are increasingly pervasive in contemporary societies across numerous countries, including China [3]. Materialistic values (or materialism) refer to a value orientation that emphasizes the importance of material wealth in pursuit of happiness and successful life. Specifically, materialistic values can be viewed of as consisting of three facets, including (1) possession-defined success, reflecting the view that some of the most important achievements in life include acquiring material possessions; (2) acquisition centrality, indicating that people like a lot of expensive things in their lives; and (3) the acquisition as the pursuit of happiness, for example, people thinking they would be happier if they could afford to buy more things [4]. Based on this conceptualization, the Material Values Scale was developed to measure materialistic values among our participants [4].

Dynamic changes in the modern society have led to social mobility and probably a confusion in norms, increasing a sense of personal insecurity [5]. Researchers have suggested that the accumulation of material wealth is an effective way compensating for insecurity [5]. This noted, materialistic values also have a negative relationship with mental health and personal well-being, such that higher dispositional materialistic orientation or primed materialism was found to be related to lower individual well-being, including risky health behaviors, negative self-evaluations, and pronounced emotional problems, etc. [6, 7]. Moreover, some other studies have revealed that materialistic values are also related to societal well-being and social problems [7], such as compulsive purchases [8], environmental damage [9], reduced pro-social behavior [10], and even unethical behaviors [11]. Therefore, identifying potential factors that underlie the formation of materialism has vital theoretical and practical values.

It is widely recognized that the external environment plays a crucial role in shaping individuals’ internal values and beliefs, including materialistic values [12]. The messages conveyed through media, advertising, and popular culture have a profound impact on individuals. These mediums often promote certain lifestyles, values, and consumerism, affecting how people perceive success, happiness, and societal norms [13]. We posit that frequent exposure to information disseminated through social network sites (SNSs) is correlated with individuals’ heightened aspiration for material wealth. This is because “broadcast” on SNSs (e.g., Twitter, Facebook, WeChat posts and advertisements) is often saturated with messages that promote consumerism and materialism, equating happiness and success with wealth and consumption [13]. Indeed, there is some empirical evidence has revealed that SNS use is associated with materialistic value orientation [14, 15]. However, less is known about the possible mediating and moderating mechanisms linking SNS use and materialistic values to important self-related processes, in particular the psychological processing that influences the formation of self-control and self-acceptance [16]. These are crucial factors for understanding online behavior [17, 18]. We focus on self-control and self-acceptance because both of these self-related processes are important for young people’s psychological functioning [19], and are likely to help them curb temptations on material consumptions otherwise saturated on SNSs [15, 20]. Thus, we propose that studying self-processes such as self-control and self-acceptance can provide valuable insights for understanding the mechanism and boundary conditions of the effects of SNS use and materialistic values on people’s responses. Below we reflect on possible connections between these variables.

### SNS use and materialistic values

SNS use has shown explosive growth worldwide. People now have easy and instantaneous opportunities to exchange information and keep connection with others due to the SNS tools. For example, in China, WeChat is the most popular SNS, with approximately 1.225 billion monthly active users, growing at a rate of 5.2% year-by-year, according to its developer Tencent’s 2020 financial report (https://www.tencent.com). Like many other popular SNSs (e.g., Facebook, Instagram, and Twitter), people can easily post information (e.g., update status, post pictures, share contents) and interact with others (e.g., comment, “like”, or directly chat) on WeChat, which help them make self-disclosure and manage their self-image [21]. At the same time, this virtual environment may have the potential to promote social comparison, imitation, jealousy, and other negative outcomes such as anxiety, depression and lower self-evaluation [22–27].

Furthermore, active or passive use of SNS can also have different effects on users’ mental health [13, 28]. For example, compared with active SNS use (e.g., posting status), passive SNS use (e.g., lurking on others’ pages) was found undermined user’s affective well-being through heightened envy [29]. Ozimek and Bierhoff [24] also found that passive Facebook use was associated with lower self-esteem and higher depressive tendencies, and these associations were mediated by higher social comparison orientation.

Uses and Gratifications Theory suggested that social media platforms offer people a chance to actively seek out specific contents for meeting their personal needs and goals (such as obtaining information, entertainment, social interaction, self-expression, emotional satisfaction, etc.), rather than just passively accepting media contents [30]. Furthermore, Social Online Self-Regulation Theory (SOS-T) proposes that social media has the potential for self-regulation, functioning as a tool to achieve diverse goals such as self-presentation (including materialistic aspiration), social interaction, and more [13]. Ultimately, this facilitates the realization of hedonically positive outcomes (e.g., good feelings, happiness). In other words, SNSs can be a powerful tool for users pursuing material enjoyment, displaying possessions and consumptions for meeting their materialistic-related desires and needs relevant to self-presentation. In line with the SOS-T, research has found that higher measured or primed materialism is associated with increased use of SNS [31, 32].

SOS-T and associated models [31, 32] have mainly focused on how users’ materialistic value orientation (as personality characteristic) predicts the consumption on SNS. Building and extending on SOS-T, we argue that it is also possible that frequent exposure to (i.e., an environmental factor) or use of SNS (i.e., a behavior) may deepen the material values adopted by users [15]. The occurrence of this phenomenon is attributable to the current prevalence of consumer culture elements in content showcased on SNSs, encompassing brands, luxury goods, fashion models, and celebrity worship [33, 34]. The exposure to these components holds the capacity to profoundly shape users’ perspectives on material possessions and wealth [35]. This influence is particularly conspicuous among young users, who exhibit an increased vulnerability to media impact [36].

SNSs now are conveying much consumption information that may explicitly or implicitly emphasizes the possession of material wealth [37]. For instance, there are plenty of business advertisements displayed in WeChat Moments/Loops or by the commercials’ official accounts. Compared to TV commercials, the content displayed on SNS posters (e.g., brand operators, advertisements) is typically more finely crafted and precisely targeted to cater to users’ interests and needs [38].

Moreover, “friends” on the user’s list may often post their shopping or tourism information which might impress others that more material wealth brings happier lives. As Ferguson and Kasser [39] have suggested, commercial media exposures are closely linked to materialistic aspirations. Indeed, a study by Chan and Prendergast [35] found that motivation for viewing advertisements was positively associated with imitation of celebrity models, which, in turn, positively predicted Chinese youths’ adoption of materialistic values. Ho’s study found that consumption-oriented SNS use, including access to marketing messages and communication with peers on consumption activities, positively predicted young users’ materialistic values through elevated perception of peers’ consumption level [15]. Furthermore, even when not considering the content of SNS use, research has identified a positive relationship between smartphone addiction and adolescent materialism [40]. Moreover, excessive SNS usage has been found to be positively associated with young adults’ online compulsive buying, a manifestation of materialistic behavior [8].

In light of these findings, we put forward our first hypothesis.

- H1: The intensity of SNS use is positively correlated with the adherence to materialistic values.

### Self-control links SNS use with materialistic values

Self-related process can be a crucial factor in understanding online behavior because it influences the formation of goal orientation and behavioral habits in social networks [18, 41, 42]. Self-related variables are also important factors of internalizing and externalizing problems (e.g., compulsive buying and substance abuse), with spontaneous and initiative power influencing our behaviors and values [43, 44]. In particular, self-control is an important goal-directed ability in the self-processes, referring to the capacity to change inherent or habitual behavior and maintain long-term goals by overcoming inner desires and external temptations [45]. It is worth noting that, in the Social Online Self-Regulation Theory (SOS-T) [13], self-regulation, a construct closely related to self-control [46], is treated as a central process that can potentially encompass all other motives and goals, and is conceptualized to explain why people use SNSs. For instance, according to a self-regulatory perspective, SNS usage is treated as a *means* for materialists to reach their *sub-goal*, such as an increase in material possessions (e.g., more digital friends), which eventually meets their desired end state such as happy life [31, 32]. In this context, self-regulation can be viewed as a comprehensive concept encompassing all goal-directed processes. This can include self-control, which primarily denotes the capability to facilitate these goal-directed processes.

Despite SOS-T has been proposed mainly for explaining why people use SNSs [31, 32], it also indicates that this kind of self-regulation strategy can be actually dysfunctional and bring unhappiness, for example, frequent SNS use was found correlated with negative outcomes such as lower self-esteem and higher depressive tendencies [22, 24]. Next, we specify how SNS use may link to materialistic values through self-control.

As a central capacity for goal achievement, high self-control is found positively associated with social adaptation, physical and mental health, such as life satisfaction and happiness [45, 47]. Conversely, individuals with low self-control are less tolerant of uncertainty [48], and are more likely to exhibit deviant behaviors, such as internet addiction [44], eating disorders [49], antisocial behaviors [50], and excessive consumption [51, 52]. In this way, self-control may be negatively associated with materialistic value orientation [53].

As a relatively stable trait, one can say that lower self-control capacity may be associated with overuse and indulging in SNSs (i.e., personality→behavior) [54, 55]. It is also possible, however, that a frequent use tendency of SNS may be related to much depletion on one’s overall self-control capacity (i.e., behavior→personality), as has been suggested by the Strength Model of Self-control [56]. This model notes that self-control capacity relies on the limited cognitive resources, which will be consumed when one is performing activities such as selective attention, emotional management, and behavioral control. This state may impair other subsequent behaviors that also need self-control resources.

In the digital era, explosive messages and temptations are prevalent on the social media than before. Even though this kind of information brings users short-term enjoyment, it also increases the burden of information identification and selection, and distracts users’ attention on the task at hand. In short, the engagement of social media can consume users’ cognitive resources, leading to impulse or self-control failure. Empirical research has revealed that students who use SNS (e.g., Facebook) longer are more likely weaker in self-control [57]. And even a 5-minute “dose” of SNS use can significantly lower the user’s self-control in the subsequent choice tasks [58]. Similarly, individuals who have problematic SNS usage are more likely to fail at exerting self-control [59].

Taken together, given that SNS use intensity is closely correlated with self-control [57, 60, 61], which may be negatively associated with materialistic values [53], we present the next hypothesis.

- Hypothesis 2: Self-control may mediate the association between SNS use intensity and materialistic values.

### Self-acceptance moderates the associations of SNS use, materialistic values, and self-control

Importantly, despite SNS use intensity is potentially associated with lower self-control and stronger materialistic values, these associations may not be applicable to all users, because some protective factors such as self-acceptance may help buffer the unfavorable associations of SNS use with them.

Self-acceptance refers to a tendency to live with one’s actual self and make decisions in terms of their inner needs and true beliefs, instead of evaluating themselves based on external standards [62]. High self-acceptance individuals tend to appreciate their own strengths but also realize and accept their weaknesses. They clearly know the gap between their actual and ideal selves and are genuinely willing to progress for the ideal self. Relevantly, self-esteem refers to a positive evaluation and emotional value attributed to oneself [63]. Self-acceptance and self-esteem are interconnected in terms of self-perception and emotional evaluation [64], but self-acceptance may provide the foundation for individuals to embrace themselves and thus contribute to the cultivation of self-esteem [65].

The social compensation hypothesis has suggested that people who are less acceptance of themselves tend to believe that SNSs can compensate for offline social and personal problems [60]. Therefore, they may spend more time in online communication and presentation, in order to gain the positive external evaluation and create a positive image of the self, which could increase the risk of internet indulgence, deplete cognitive resources and reduce the level of self-control in other domains [61]. On the contrary, high self-acceptance individuals have optimal self-evaluation and have the “ownership” of their life [62], so they need not satisfy their positive image by indulging and using up too much resources in the virtual network, thus palliating its impairment on self-control. Therefore, as a protective factor, high self-acceptance may moderate the negative association of SNS use and self-control. We then present the next hypothesis as follows.

- Hypothesis 3: Self-acceptance may moderate the relationship between SNS use intensity and self-control.

Likewise, since individuals with high self-acceptance may clearly know their actual needs and evaluate themselves based on inner standards, they thus do not need to excessively consume due to external reasons such as social comparison [62], in other words, they may generally pay less attention to and deal better with the material and consumption information shared on social media. Due to the relative lack of literature reflecting on self-acceptance and materialistic orientation, we look at self-esteem for a reference. When individuals’ self-esteem needs are not adequately met, they often resort to excessive pursuit of external material possessions as a means to compensate for their sense of value and fulfillment [66]. Material wealth serves as a compensatory function for individuals’ self-esteem [67]. This viewpoint is supported by a substantial body of empirical evidence. For instance, individuals with lower self-esteem have been found to exhibit higher materialistic values [68, 69]. As noted earlier, embracing self-acceptance enables individuals to acknowledge their weaknesses and foster tolerance for self-evaluation, even when it tends to be less favorable [65]. This, in turn, may alleviate the inclination towards materialistic values as a means of compensating for low self-esteem. Consequently, the cultivation of self-acceptance could potentially temper one’s inclination towards materialism, thereby weakening the correlation between SNS use and the pursuit of materialistic values.

Therefore, a fourth hypothesis is presented as follows.

- Hypothesis 4: Self-acceptance may moderate the relationship between SNS use intensity and materialistic values.

### The present study

**Fig. 1:**
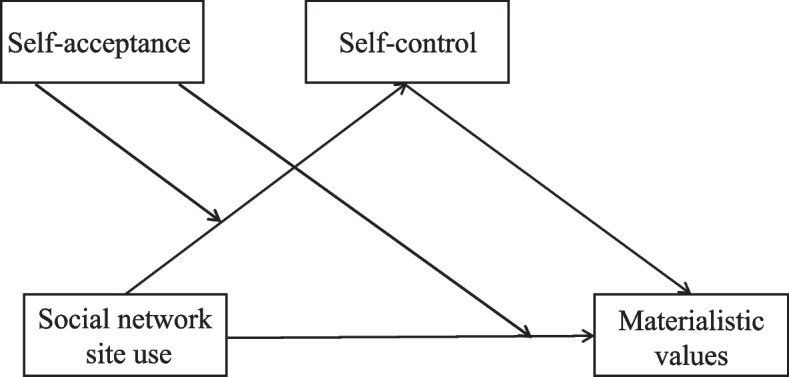
The proposed theoretical model

The aims of the present study are to examine (a) whether self-control can mediate the relationship between SNS use intensity and materialistic values, and (b) whether self-acceptance would moderate the association between SNS use intensity and materialistic values, as well as the association between SNS use intensity and self-control. We formulate the moderated mediation model (Fig. 1) to unravel these above relationships.

## Methods

### Participants

An a priori power analysis suggested that approximately 190 participants were needed to detect a bivariate correlation (*r* = 0.20) among the main variables with sufficient statistical power of 0.80 (α = 0.05, two-tailed), comparable to findings obtained in earlier research on similar topics [40, 55]. We enlarged this sample size for detecting potential mediation or moderation effects. In the end, 706 university students (381 women, 279 men, and 46 did not fill in gender; *M* = 18.15, *SD* = 0.67, age spanning from 16 to 21) from Shandong Province, China were recruited as participants. Continuous average was used to handle items missing data [70]. All participants reported having experience of using WeChat.

### Procedure

Before questionnaire administration, the researchers acquired informed consent from the participants. Each participant was given a self-report questionnaire in the classroom and completed the items independently in approximately 20 minutes. Participants were assured that they were free to quit at any time as they wish. This research was in accordance with the 1964 Helsinki Declaration and approved by the Human Research Ethics Committee of the first author’s institution.

## Measures

### SNS use

SNS use was assessed by the intensity of WeChat use scale which was adapted from the Facebook Intensity Scale [71] with slight wording modifications (i.e., replacing “Facebook” with “WeChat”). This scale was validated in Chinese [72] and showed satisfactory reliability and validity in previous research [18]. It consists of 3 parts, including 6 items in total. The first part includes four questions (e.g., I feel out of touch when I haven’t logged onto WeChat for a while) with a 5-point scale, ranging from 1 (*very strongly disagree*) to 5 (*very strongly agree*). The second part (i.e., the fifth question) asks “How many friends are there on your WeChat?”. This item was rated on a nine-point scale (0 = *10 and below*; 1 = *11–50*; 2 = *51–100*; 3 = *101–150*; 4 = *151–200*; 5 = *201–250*; 6 = *251–300*; 7 = *301–400*; 8 = *more than 400*). The third part (i.e., the sixth question) asks “How many minutes do you use WeChat every day on average?” This item was rated on a six-point scale (0 = *less than 10 minutes*; 1 = *10–30 minutes*; 2 = *31–60 minutes*; 3 = *1–2 hours*; 4 = *2–3 hours*; 5 = *more than 3 hours*). As the scoring ranges in three parts are different, all items were first standardized before doing the following analysis [71]. Higher standardized mean score indicates a high degree of SNS use and emotional involvement. The scale revealed a satisfactory internal consistency in the current sample (Cronbach’s α = 0.74).

### Materialistic values

The Material Value Scale was compiled by Richins and Dawson [4] and the Chinese version was revised by Li and Guo [73]. The revised scale consists of 13 items and includes possession-defined success, acquisition centrality, and acquisition as the pursuit of happiness. The items were rated on a five-point scale from 1 (*strongly disagree*) to 5 (*strongly agree*). The higher averaged score of all items represents the higher level of materialistic orientation [73]. In this study, Cronbach’s α for this questionnaire was 0.79.

### Self-control

The Trait Self-Control Scale was compiled by Tangney et al. [45] and was later revised in Chinese by Tan and Guo [74]. The revised scale included 19 items such as (1) impulse control (e.g., I lose my temper too easily), (2) keeping healthy habits (e.g., I have a hard time breaking bad habits), (3) inhibiting temptation (e.g., I am good at resisting temptation), (4) focusing on work (e.g., I am able to work effectively toward long-term goals), and (5) controlling entertainment (e.g., I spend too much money). The scores of the scale ranged from 1 (*not at all characteristic of me*) to 5 (*entirely characteristic of me*). Higher mean scores of all items indicate a stronger capability for self-control [74]. The Cronbach’s alpha coefficient of the total scale was 0.85 in this study.

### Self-acceptance

The Self-acceptance Questionnaire (SAQ) was compiled by Cong and Gao [75], and had been widely used in previous research [76]. The questionnaire consists of 16 items, including self-evaluation dimension (e.g., Generally speaking, I am very satisfied with myself) and self-acceptance dimension (e.g., I am often concerned that people look down on me), ranging from 1 (*completely disagree*) to 4 (*completely agree*). The higher mean score (reverse-scored items were converted) represents better degree of self-acceptance [75]. This scale revealed good internal consistency (Cronbach α = 0.80) in the current sample.

### Control variables

Since prior research have revealed gender and age differences in SNS use intensity [18], self-control [77] and materialistic orientation [78], age and gender were thus treated as control variables in the main analyses. Besides, socioeconomic status (SES) was found correlated with self-control [79] and materialism [80], therefore, SES was also controlled in the main analyses. Gender was dummy coded with 1 = man, and 2 = woman. SES score was calculated by averaging the standardized scores of educational level and monthly income of both parents. Parental educational level was coded from 1 (equal to or below junior high school) to 4 (graduate education or above). The mean values for paternal and maternal educational level were 1.67 (*SD* = 0.77) and 1.49 (*SD* = 0.70), respectively. The monthly parental income was coded from 1 (< 2000 yuan) to 5 (> 5000 yuan). The mean monthly incomes for fathers and mothers were 3.49 (*SD* = 1.38) and 2.48 (*SD* = 1.38), respectively.

### Data analysis

We used IBM SPSS 21.0 for data processing and analyses. First, preliminary analyses were conducted to show the means, standardized deviations and correlations among the main variables. Second, the PROCESS macros in SPSS [81] were used to examine the proposed mediating and moderating effects. Bootstrap confidence intervals (CIs) were used to determine whether the mediating/moderating effects were significant based on 5000 random samples [81]. An effect would be regarded as significant if the CIs did not include zero. The main variables were standardized for reducing multi-collinearity before doing the mediation or moderation analyses [82].

## Results

### Preliminary analyses

Descriptive statistics and correlation matrix of SNS use intensity, materialistic values, self-control, and self-acceptance are illustrated in Table 1. As the results showed, SNS use intensity was positively associated with materialistic values, but was negatively associated with self-control. Materialistic values were negatively associated with self-control and self-acceptance. And self-control was positively associated with self-acceptance. No significant correlation was found between SNS use intensity and self-acceptance.

**Table 1:** Means (*M*), Standard Deviations (*SD*) and Pearson correlations among the main variables

Variables	*M*	*SD*	1	2	3	4
1. SNS use intensity	0.00	0.66	–			
2. Materialistic values	2.67	0.58	0.227**	–		
3. Self-control	3.26	0.58	−0.121**	−0.401**	–	
4. Self-acceptance	2.43	0.40	0.048	−0.251**	0.373**	–

**p < 0.010. The mean score for SNS use intensity was standardized, while mean scores for other variables were retained in their raw form

### Testing for the mediation model

To examine whether self-control could mediate the relationship between SNS use intensity and materialistic values, we conducted mediation analysis with Model 4 in the PROCESS macro [81]. The coefficients reported in the following mediation or moderation model (including those in tables and figures) were all standardized.

As shown in Table 2, after controlling for gender, age and SES, the direct path coefficient from SNS use intensity to materialistic values was significant (*β* = 0.34, *p* < 0.001). In addition, SNS use intensity was negatively associated with self-control (*β* = − 0.17, *p* < 0.01). When SNS use intensity and self-control were simultaneously included as predictors of materialistic values, self-control was negatively associated with materialistic values (*β* = − 0.38, *p* < 0.001), and SNS use intensity was positively correlated to materialistic values (*β* = 0.28, *p* < 0.001). That is, higher SNS use intensity was associated with lower self-control, which was correlated to stronger adherence to materialistic values. Moreover, the bias-corrected percentile bootstrap method revealed that the indirect effect of SNS use intensity on materialistic values via self-control was significant (indirect effect = 0.06, *SE* = 0.02, 95% CI = [0.02, 0.11]). And the mediation effect accounted for 19% of the total effect of the association between SNS use intensity and materialistic values.

**Table 2:** Testing the mediation effect of SNS use intensity on materialistic values via self-control

	*Outcome* (Materialistic values)		*Outcome* (Self-control)		*Outcome* (Materialistic values)	
***Predictors***	*β*	*t*	*β*	*t*	*β*	*t*
Age	−0.08	−2.15*	0.02	0.63	−0.07	−2.07*
Gender	−0.01	−0.13	− 0.03	−0.86	− 0.02	−0.51
SES	−0.01	−0.27	− 0.03	−0.89	− 0.03	−0.66
SNS use intensity	0.34	6.06***	−0.17	−2.94**	0.28	5.30***
Self-control					−0.38	−11.08***
*R*^*2*^	0.058		0.018		0.198	
*F*	10.74***		3.14*		34.65***	

^⁎^ *p* < 0.050, ^⁎⁎^ *p* < 0.010, ^⁎⁎⁎^ *p* < 0.001. The term “Materialistic values” mentioned first in the table denotes the direct effect under examination, while the second occurrence of “Materialistic values” pertains to the tested mediation effect

### Moderated mediation effect analysis

To test the moderated mediation model, we used Model 8 in the SPSS PROCESS macro [81]. Specifically, we estimated the moderating effects of self-acceptance on the relationship between SNS use intensity and self-control, and on the relationship between SNS use intensity and materialistic values.

As shown in Table 3, when materialistic values were treated as the dependent variable, after controlling for gender, age and SES, SNS use intensity was positively correlated with materialistic values (*β* = 0.29, *p* < 0.001), but the interaction of SNS use intensity×self-acceptance on materialistic values was not significant (*β* = 0.01, *p* = 0.789). Therefore, it seems that self-acceptance did not moderate the relationship between SNS use and materialistic values.

**Table 3:** Testing the moderated mediation effects

	*Outcome* (Self-control)		*Outcome* (Materialistic values)	
***Predictors***	*β*	*t*	*β*	*t*
Age	0.01	0.23	−0.07	−2.06*
Gender	−0.01	−0.19	− 0.03	−0.75
SES	−0.09	−2.50*	−0.004	− 0.11
Self-acceptance	0.40	11.41***	−0.14	−3.76***
SNS use intensity	−0.20	−3.75***	0.29	5.64***
Self-control			−0.32	−8.80***
Self-acceptance × SNS use intensity	0.14	2.66**	0.01	0.27
*R*^*2*^	0.174		0.215	
*F*	24.58***		27.30***	

^⁎^ *p* < 0.050, ^⁎⁎^ *p* < 0.010, ^⁎⁎⁎^ *p* < 0.001

**Fig. 2:**
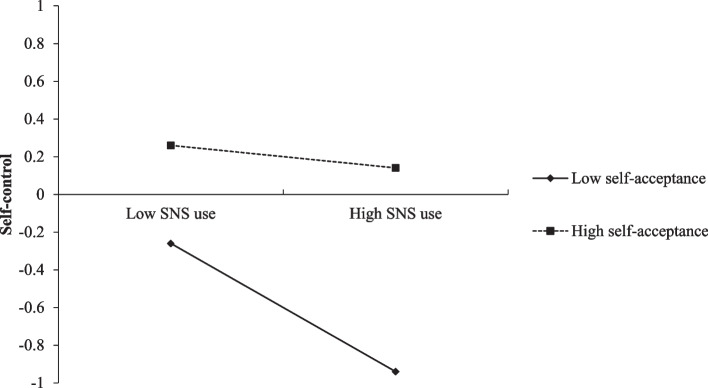
Interaction of SNS use intensity and self-acceptance on self-control

Furthermore, when self-control was treated as the dependent variable, as shown in Table 3, after controlling for gender, age and SES, SNS use intensity was negatively correlated with self-control (*β* = − 0.20, *p* < 0.001), and the interaction effect of SNS use intensity×self-acceptance on self-control was also significant (*β* = 0.14, *p* < 0.01). To better understand the moderating effect of self-acceptance, we conducted simple slope tests and plotted the relationship between SNS use intensity and self-control separately at low (1 *SD* below the mean) and high levels (1 *SD* above the mean) of self-acceptance (Fig. 2).

The results revealed that for individuals with low self-acceptance (1 *SD* below the mean), SNS use intensity was significantly and negatively associated with self-control, *β* = − 0.34, *p* < 0.001. However, for high self-acceptance individuals (1 *SD* above the mean), the relationship between SNS use intensity and self-control became non-significant, *β* = − 0.06, *p* = 0.452.

**Fig. 3:**
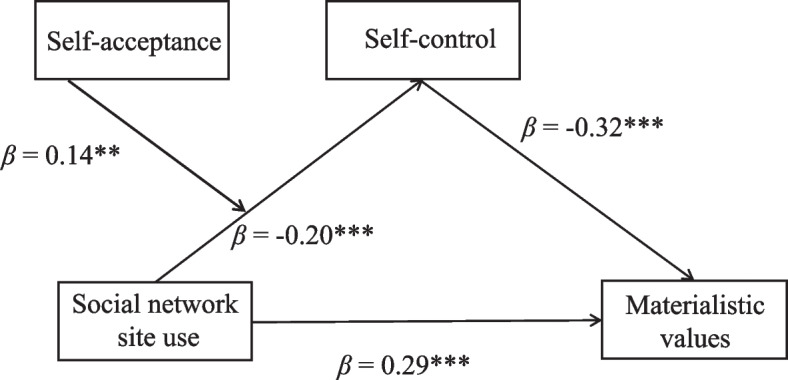
The final tested moderated mediation model

Furthermore, bias-corrected percentile bootstrap analyses showed that the indirect effect of SNS use intensity on materialistic values via self-control was moderated by self-acceptance. Specially, for low self-acceptance individuals, the indirect effect of SNS use intensity on materialistic values via self-control was significant, *β* = 0.11, *SE* = 0.03, 95% CI = [0.05, 0.17]. But for high self-acceptance individuals, this indirect effect was not significant, *β* = 0.02, *SE* = 0.02, 95% CI = [− 0.03, 0.07]. In sum, these results confirmed the moderated mediation model (Fig**.** 3) and indicated that self-acceptance moderated the indirect effect of SNS use intensity and materialistic values via self-control.

### Testing of alternative models

**Fig. 4:**
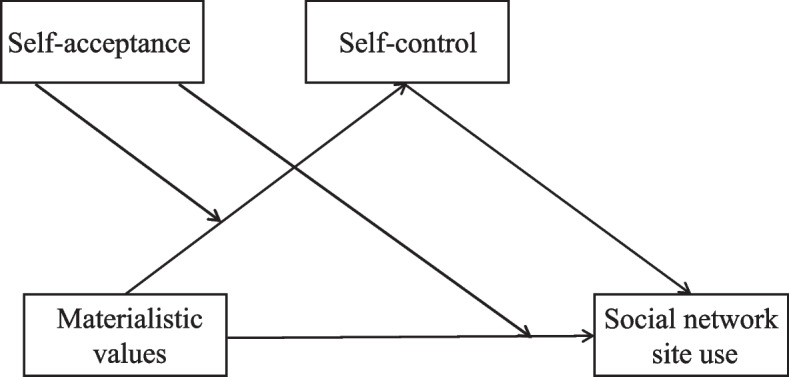
The illustration of alternative model A

**Fig. 5:**
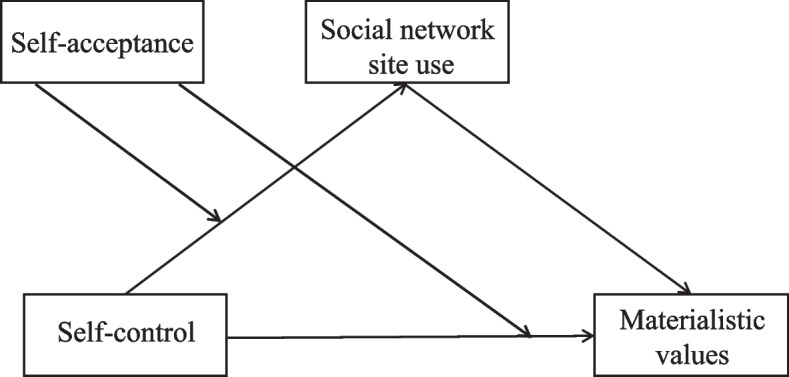
The illustration of alternative model B

Although we proposed a moderated-mediation model to explain the effect of SNS use on materialism through the mediator of self-control and the moderator of self-acceptance, there might be other models that may also fit the data. Thus, given the correlational quality of the data, and based on earlier research, it is important to consider two alternative models. As depicted in Fig. 4, in Alternative Model A, based on SOS-T [13], SNS use and materialistic values may switch their positions, such that materialistic values may be treated as a predictor for SNS use. While in Alternative Model B (in Fig. 5), as a relatively stable capacity, poor self-control may be an antecedent of frequent SNS use [55], which is sequentially associated with materialistic values.

Specifically, in Alternative Model A, higher materialistic value orientation may link to more frequent use of SNS via the mediating role of self-control, and self-acceptance may moderate these associations. As suggested by the SOS-T [13], social media platforms function as a tool for achieving one’s hedonic-related goals including materialistic aspiration. In this context, higher materialistic orientation would be associated with frequent use of SNS [31, 32].

Furthermore, individuals who affiliate more strongly with materialistic values have relatively lower construal level, indicated by emphasizing concrete, subordinate considerations, such as immediate desires and impulses which are known to be associated with weaker self-control [48, 83]. These variables are known to be related with people becoming more impulsive and disinhibited, which in turn is related to being more prone to social media addiction or problematic SNS use [54]. In this way, self-control may link materialistic values with SNS use and mediates their association. In addition, individuals who are lower in self-acceptance may rely more on external material wealth to showcase their self-image and compensate for their sense of value and fulfillment [66], which may further exacerbate materialism’s effects on other outcomes such as self-control and SNS use. Therefore, it is possible that the above mediating paths are moderated by self-acceptance.

We first conducted a mediation analysis (after controlling for gender, age and SES; the same after) to test the mediating role of self-control. To maintain conciseness, detailed information on mediation and moderation analyses for Alternative Models A and B can be found in the supplementary material tables. Results revealed that materialistic values were negatively associated with self-control (*β* = − 0.40, *p* < 0.001) and positively associated with SNS use intensity (*β* = 0.14, *p* < 0.001), while self-control was not significantly associated with SNS use intensity (*β* = − 0.02, *p* = 0.523). The bias-corrected percentile bootstrap method confirmed that the indirect effect of materialistic values on SNS use via self-control was not significant (indirect effect = 0.007, *SE* = 0.01, 95% CI = [− 0.02, 0.03]). These results suggested that self-control did not mediate the relationship between materialistic values and SNS use, when materialistic values were treated as a predictor.

**Fig. 6:**
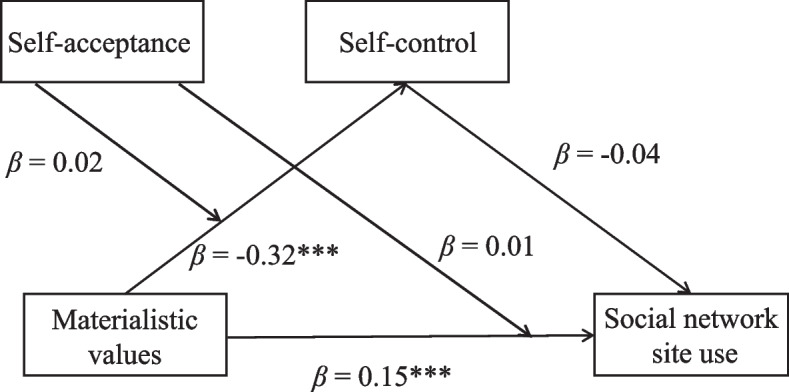
The results of alternative model A

We then tested the moderation role of self-acceptance. When self-control was treated as the dependent variable, materialistic values were negatively correlated with self-control (*β* = − 0.32, *p* < 0.001), but the interaction effect of materialistic values×self-acceptance on self-control was not significant (*β* = 0.02, *p* = 0.52). When SNS use was treated as the dependent variable, materialistic values were positively correlated with SNS use (*β* = 0.15, *p* < 0.001), but the interaction effect of materialistic values ×self-acceptance on SNS use was also not significant (*β* = 0.01, *p* = 0.51). Therefore, it seems that self-acceptance did not moderate the associations between materialistic values and self-control, as well as between materialistic values and SNS use. In other words, the moderated mediation model (Alternative Model A) is not supported. See Fig. 6 for a reference.

Regarding Alternative Model B, failure in self-control may be one of the antecedents of frequent or problematic SNS usage, as suggested in previous research [44, 54, 55]. As we have constructed in the introduction, self-control failure is also relevant to impulsive consumption behaviors such as excessive and compulsive buying [51, 52], a manifestation of materialism. In this way, lower self-control may be associated with higher SNS use intensity, which further mediates the relationship between self-control and materialistic values. Also, self-acceptance may moderate the relevant paths. For example, individuals with lower self-acceptance might be more likely to seek a sense of existence and satisfaction from the external sources such as social media platforms, which can make it more difficult to deploy self-control against the online temptations [61, 62]. In other words, as crucial components in self-processes influencing psychological functioning [16], self-acceptance may impact the role of self-control in various outcomes, including SNS use and materialistic values. This is plausible since self-control may see improvement when individuals are motivated to acknowledge their mistakes and accept themselves [84]. Therefore, self-acceptance may moderate the associations regarding how self-control links to SNS use and materialistic values.

We first conducted the mediation analysis. Results revealed that self-control was negatively correlated to intensity of SNS use (*β* = − 0.07, *p* < 0.01). When SNS use intensity and self-control were simultaneously included as predictors of materialistic values, self-control was negatively associated with materialistic values (*β* = − 0.38, *p* < 0.001), and SNS use intensity was positively associated with materialistic values (*β* = 0.28, *p* < 0.001). Moreover, the bias-corrected percentile bootstrap method revealed that the indirect effect of self-control on materialistic values via SNS use intensity was significant (indirect effect = − 0.02, *SE* = 0.009, 95% CI = [− 0.04, − 0.01]), suggesting that SNS use intensity mediated the relationship between self-control and materialism, when self-control was treated as a predictor.

**Fig. 7:**
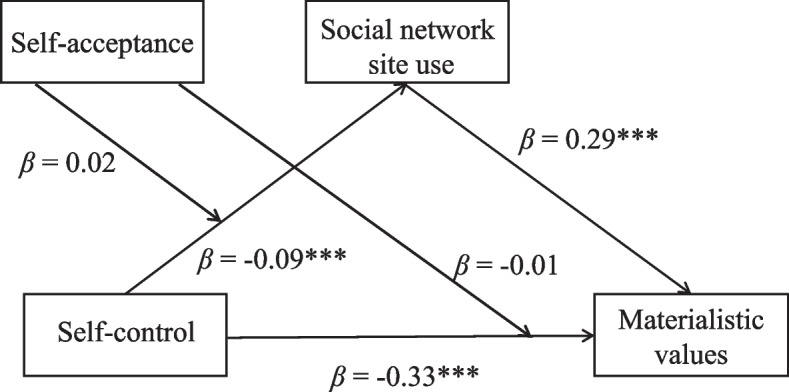
The results of alternative model B

Next, we tested the moderated mediation model. As shown in Fig. 7, when SNS use intensity was treated as the dependent variable, self-control was negatively correlated with SNS use (*β* = − 0.09, *p* < 0.001), but the interaction effect of self-control×self-acceptance on SNS use intensity was not significant (*β* = 0.02, *p* = 0.42). When materialistic values were treated as the dependent variable, self-control was negatively correlated with materialistic values (*β* = − 0.33, *p* < 0.001), while the interaction effect of self-control×self-acceptance on materialistic values was also not significant (*β* = − 0.01, *p* = 0.63). Therefore, it seems that self-acceptance did not moderate the relationships between self-control and materialistic values, as well as between self-control and SNS use. That is, the moderated mediation model (Alternative Model B) is also not supported.

In view of different models, it seems that our hypothesized model may be better supported by the data. We next discuss how the main findings should be understood.

## Discussion

The potential influence of SNS use on materialistic values has acquired much theoretical and empirical support, in particular for young people [15, 39]. However, the mediating and moderating mechanisms underlying this association, particularly from the perspective of self-processes, remain largely unknown. The current research puts forward a moderated mediation model. In line with this model, our results suggest that self-control partially mediates the relation between SNS use intensity and materialistic values. Furthermore, this mediation effect is moderated by self-acceptance. Overall, these pathways can extend our knowledge of the mechanisms underlying the relationship between SNS use intensity and materialistic values and provide promising practical guidance for reducing the adverse effects of SNS use on adherence to materialistic values.

### Explanation of the relations among SNS use, materialistic values, and the mediating role of self-control

The association of SNS use and materialistic values has been extensively explored by much research [8, 14, 15, 40]. In particular, from a self-regulatory perspective, the SOS-T [13] suggested that social media platforms could serve as a useful medium for pursuing one’s goals such as self-presentation and related materialistic aspiration, in this way, materialists were found used the SNSs more frequently [31, 32]. Despite SOS-T have mainly focused on how materialistic orientation predicts SNS use (i.e., personality characteristic→behavior) [31, 32], it also warned that the use of SNS tools can actually bring dysfunctional outcomes such as depression tendencies and lower self-evaluation [22, 24]. Our mediation model confirms that more frequent use of SNS may be associated with self-control failure and even higher materialistic value orientation, supporting SOS-T’s arguments [13], and further suggests that the association of materialistic values and SNS use may be bi-directional. Of course, this assumption needs further causal design examinations. We next focus on how the mediating process should be understood.

Our results reveal that self-control is a potential self-related mechanism that helps explain why more SNS usage is associated with stronger adoption of materialistic values. Considering that prior investigations on the mechanisms of SNS use and materialistic values have mainly focused on the “external” factors such as social comparison, peer and parent-child relationships [15, 40, 85], the present research might be valuable in uncovering the “inner” self-related process, which may be much closer to one’s more stable values.

On the one hand, it may be noted that long-time engagement on social media activities can lead to cognitive load and deplete the limited psychological resource, further reducing self-control capacity [56]. Consequently, individuals with reduced self-control ability might have problem resisting material temptations and comparisons, leading to higher consumerism and materialistic values.

On the other hand, it can also be argued on the basis of the Hot/Cool-System Framework [86], that individuals who are obsessed with social media might be more likely to suffer from emotional distress as a result of their tendencies to activate the hot, emotional “go” system, and inhibit the cool, cognitive “know” system. If this line of reasoning would have merited, then, high intensity SNS users may be more emotionally impulsive, have poorer self-control ability, and exhibit more materialistic-related problematic behaviors such as material addiction and excessive consumption.

### Explanation of the moderating role of self-acceptance

We note explicitly that an important issue is whether there are protective factors that may buffer the negative influences of SNS use intensity on self-control and materialistic values. Our results revealed that the indirect path from SNS use to materialistic values through self-control was moderated by self-acceptance. Specifically, the negative effect of SNS use intensity on materialistic values via self-control was significant only for low (but not high) self-acceptance individuals, suggesting the protective role of self-acceptance. Earlier research revealed that users of SNS with a larger online friend circle tend to exhibit increased materialism, attributed to heightened motivation for extrinsic goals such as wealth and status. Prior studies also found that self-esteem can serve as a mitigating factor against these adverse effects of SNS use [85]. Expanding on these findings and in alignment with prior research [85], our study underscores the protective roles of both self-esteem and self-acceptance in alleviating the negative impact of SNS use on individuals’ materialistic values. Furthermore, our investigation delves into the mediating and moderating influences of self-control and self-acceptance. This approach aims to deepen our comprehension of the underlying self-processes that shape the relationship between SNS usage and personal values.

Consistent with Social Compensation Theory [87], individuals with low self-acceptance may prefer to present themselves or satisfy their social needs through online compared to offline, due to social media’s characteristics of asynchronous communication and anonymity. This tendency may increase the risk of problematic behaviors such as SNS overuse and addiction, and subsequently damaging self-control capacity. Similar to individuals with low self-esteem, those who perceive a lack of offline social interactions may believe that SNS can compensate for this deficiency by spending more time engaging in online communication and self-presentation, but face the risk of losing control of SNS use and even internet addiction [88, 89].

Conversely, high self-acceptance individuals may better know their actual needs and thus use social media in a more rational and healthy manner. Consistent with Rich-get-richer Theory [60], individuals with high self-acceptance may have better social communication ability and skills to get positive feedback they need (e.g., social support, useful information) from the social media. The accumulated positive experiences/resources may give users a sense of control over their environment [90], helping to mitigate the negative effect of SNS use intensity on self-control.

Partly inconsistent with our expectation, however, we did not find evidence that the direct effect of SNS use intensity on materialistic values can be moderated by self-acceptance. This may imply that once one has been indulged in SNSs, the direct risk that excessive SNS use on materialistic values is difficult to be solely buffered by self-acceptance. Viewed from Self-determination Theory [91], this might be because when excessive SNS users pay much attention to extrinsic values and goals such as material success, this can be their main tool to evaluate themselves. In this case, the stable tendency to focus on extrinsic success would impede the growth of their inner goals, and perhaps is hardly addressed by self-acceptance. Accordingly, self-acceptance is less possible to moderate the direct effect of SNS use intensity on materialism. Future research is needed to further explore this issue.

### Limitations and implications

Several limitations of the current study need to be considered. First, the cross-sectional design does not permit the inference of the causality and alternative interpretations are possible. For example, the Alternative Model B found a significant mediating path of Self-control→SNS use→Materialistic values, suggesting that self-control and SNS use are likely mutually affected (see in studies which probed Self-control→SNS use [54, 55], and other studies probed SNS use Self-control [57, 58]), and they might co-work in various ways underling the formation of materialistic values. Given this, we appeal that future research should consider the longitudinal or experimental designs to further validate our moderated mediation model and test the causality of the variables’ relations. Second, the inclusion of more diverse populations may be considered in the future research, in order to test the generalization of our findings. For example, compared to college aged students, the younger adolescents’ values and self-awareness may be more easily affected by external environment such as social media due to the critical development stage, conversely, the older adults are more stable on values and self-awareness. It would thus be interesting to test whether our conceptual model is also effectual for younger and older individuals. Besides, this research focused on WeChat users in China. Considering cultural factors may impact individuals’ value orientations, attitude formation and the autonomous-related self-construal on SNS use [92], it is also necessary to test the relationship among SNS use, materialistic values and self-processes in other countries and different cultures. Third, the present study measured the general use tendency of SNS, without distinguishing its different usage types. In fact, different SNS use behaviors may have different impacts on one’s self-concept or values [18]. For instance, previous research has found that while active SNS use is positively correlated with life satisfaction, passive SNS use is related to reduced subjective well-being and increased adherence to materialistic values [27]. Investigation of different SNS use types on specific materialistic values or different self-processes might produce more promising findings.

Despite these limitations that warrant future study, we hope to have shown that the current study contributes a better understanding of how and when SNS use intensity links to materialistic values and further advance the understanding of the potential roles of self-processes. From a practical perspective, our findings may have some implications for psychological therapies or interventions for reducing the potentially negative influence of social media exposure on young people’s orientations regarding material wealth and consumption. For instance, practices of self-control skills (e.g., monitor, evaluate and reinforce one’s behavior toward a goal) [84] and establishing reasonable time limits for social media usage may help reduce exposure to and temptation to materialistic messages. It is also important to foster self-awareness and self-acceptance while directing attention towards intrinsic values, personal growth, and nurturing interpersonal relationships. By doing so, individuals can refrain from seeking fulfillment and deriving a sense of worth solely from external materialistic goals and values.

## Conclusion

In summary, our study reveals that the potentially adverse effect of SNS use intensity on materialistic values via self-control may be buffered by the protective role of self-acceptance. Our findings suggest that in the digital network era, internal self-processes still serve important roles in the formation of people’s values, suggesting that it is important to link social network site use and materialistic values through self-processes.

### Supplementary Information

**Additional file 1: Table S1. **Testing the mediation effect of alternative model A. **Table S2. **Testing the moderated mediation effects of alternative model A. **Table S3. **Testing the mediation effect of alternative model B. **Table S4. **Testing the moderated mediation effects of alternative model B.

## Data Availability

The materials, datasets, and analysis codes for the present study can be accessed through the following link [https://osf.io/hj6fv/?view_only=0970a27614b044c39950c11cd531f4c9].

## Abbreviations

SNS: Social network site
SAQ: Self-acceptance Questionnaire
SES: Socioeconomic Status
SOS-T: Social online self-regulation theory
