# Supplementary material for: Social network site use and materialistic values: the roles of self-control and self-acceptance
Source: BMC Psychol. 2024 Jan 30;12:55. doi: 10.1186/s40359-024-01546-7 (PMC10826058; doi:10.1186/s40359-024-01546-7)
Supplement: Supplementary file 1 — Additional file 1: Table S1. Testing the mediation effect of alternative model A. Table S2. Testing the moderated mediation effects of alternative model A. Table S3. Testing the mediation effect of alternative model B. Table S4. Testing the moderated mediation effects of alternative model B. [file 40359_2024_1546_MOESM1_ESM.docx]

***Supplementary Materials***

**Social network site use and materialistic values:**

**The roles of self-control and self-acceptance**

**Table S1** Testing the mediation effect of alternative model A.

|  | ***Outcome***  (SNS use intensity) | | ***Outcome***  (Self-control) | | ***Outcome***  (SNS use intensity) | |
| --- | --- | --- | --- | --- | --- | --- |
| ***Predictors*** | *β* | *t* | *β* | *t* | *β* | *t* |
| Age | 0.00 | 0.12 | -0.01 | -0.22 | 0.003 | 0.12 |
| Gender | 0.06 | 2.65** | -0.04 | -1.06 | 0.06 | 2.62** |
| SES | 0.08 | 3.47*** | -0.04 | -1.17 | 0.08 | 3.44*** |
| Materialistic values | 0.15 | 6.06*** | -0.40 | -11.52*** | 0.14 | 5.30*** |
| Self-control |  |  |  |  | -0.02 | -0.64 |
| *R²* | 0.08 | | 0.164 | | 0.077 | |
| *F* | 14.45*** | | 34.31*** | | 11.63*** | |

Note: ^⁎^ *p* < 0.050, ^⁎⁎^ *p* < 0.010, ^⁎⁎⁎^ *p* < 0.001.

**Table S2** Testing the moderated mediation effects of alternative model A.

|  | ***Outcome***  (Self-control) | | ***Outcome***  (SNS use intensity) | |
| --- | --- | --- | --- | --- |
| ***Predictors*** | *β* | *t* | *β* | *t* |
| Age | -0.01 | -0.22 | 0.003 | 0.11 |
| Gender | -0.02 | -0.54 | 0.07 | 2.81** |
| SES | -0.08 | -2.47* | 0.07 | 2.89** |
| Materialistic values | -0.32 | -9.42*** | 0.15 | 5.69*** |
| Self-acceptance | 0.30 | 8.80*** | 0.08 | 3.14** |
| Self-control |  |  | -0.04 | -1.60 |
| Self-acceptance × Materialistic values | 0.02 | 0.64 | 0.01 | 0.66 |
| *R²* | 0.247 | | 0.09 | |
| *F* | 38.25*** | | 9.83*** | |

Note: ^⁎^ *p* < 0.050, ^⁎⁎^ *p* < 0.010, ^⁎⁎⁎^ *p* < 0.001.

**Table S3** Testing the mediation effect of alternative model B.

|  | ***Outcome***  (Materialistic values) | | ***Outcome***  (SNS use intensity) | | ***Outcome***  (Materialistic values) | |
| --- | --- | --- | --- | --- | --- | --- |
| ***Predictors*** | *β* | *t* | *β* | *t* | *β* | *t* |
| Age | -0.07 | -2.09* | -0.01 | -0.30 | -0.07 | -2.07* |
| Gender | 0.0002 | 0.01 | 0.06 | 2.57* | -0.02 | -0.51 |
| SES | 0.0002 | 0.01 | 0.08 | 3.38*** | -0.02 | -0.66 |
| Self-control | -0.40 | -11.52*** | -0.07 | -2.94** | -0.38 | -11.08*** |
| SNS use intensity |  |  |  |  | 0.28 | 5.30*** |
| *R²* | 0.17 | | 0.04 | | 0.20 | |
| *F* | 34.94*** | | 7.24*** | | 34.65*** | |

Note: ^⁎^ *p* < 0.050, ^⁎⁎^ *p* < 0.010, ^⁎⁎⁎^ *p* < 0.001.

**Table S4** Testing the moderated mediation effects of alternative model B.

|  | ***Outcome***  (SNS use intensity) | | ***Outcome***  (Materialistic values) | |
| --- | --- | --- | --- | --- |
| ***Predictors*** | *β* | *t* | *β* | *t* |
| Age | -0.01 | -0.31 | -0.07 | -2.05* |
| Gender | 0.07 | 2.68** | -0.03 | -0.73 |
| SES | 0.08 | 2.99** | -0.004 | -0.11 |
| Self-control | -0.09 | -3.56*** | -0.33 | -8.83*** |
| Self-acceptance | 0.06 | 2.32* | -0.14 | -3.80*** |
| SNS use intensity |  |  | 0.29 | 5.68*** |
| Self-acceptance × Self-control | 0.02 | 0.80 | -0.01 | -0.48 |
| *R²* | 0.05 | | 0.22 | |
| *F* | 5.89*** | | 27.33*** | |

Note: ^⁎^ *p* < 0.050, ^⁎⁎^ *p* < 0.010, ^⁎⁎⁎^ *p* < 0.001.
